# Shared decision making for patients with type 2 diabetes: a randomized trial in primary care

**DOI:** 10.1186/1472-6963-13-301

**Published:** 2013-08-08

**Authors:** Megan E Branda, Annie LeBlanc, Nilay D Shah, Kristina Tiedje, Kari Ruud, Holly Van Houten, Laurie Pencille, Marge Kurland, Barbara Yawn, Victor M Montori

**Affiliations:** 1 Knowledge and Evaluation Research Unit, Mayo Clinic, 200 1st Street Southwest, Rochester, MN 55905, USA; 2 Division of Health Care Policy and Research, Department of Health Sciences Research Mayo Clinic, Rochester, MN, USA; 3 Center for the Science of Healthcare Delivery Mayo Clinic, Rochester, MN, USA; 4 Department of Anthropology and Sociology, Université Lumière Lyon 2, Lyon, France; 5 Department of Research, Olmsted Medical Center, Rochester, MN, USA; 6 Department of Family and Community Health, University of Minnesota, Minneapolis, MN, USA; 7 Division of Endocrinology Mayo Clinic, Department of Medicine, Rochester, MN, USA

**Keywords:** Decision aid, Shared decision making, Diabetes, Randomized trial

## Abstract

**Background:**

Patient-centered diabetes care requires shared decision making (SDM). Decision aids promote SDM, but their efficacy in nonacademic and rural primary care clinics is unclear.

**Methods:**

We cluster-randomized 10 practices in a concealed fashion to implement either a decision aid (DA) about starting statins or one about choosing antihyperglycemic agents. Each practice served as a control group for another practice implementing the other type of DA. From April 2011 to July 2012, 103 (DA=53) patients with type 2 diabetes participated in the trial. We used patient and clinician surveys administered after the clinical encounter to collect decisional outcomes (patient knowledge and comfort with decision making, patient and clinician satisfaction). Medical records provided data on metabolic control. Pharmacy fill profiles provided data for estimating adherence to therapy.

**Results:**

Compared to usual care, patients receiving the DA were more likely to report discussing medications (77% vs. 45%, p<.001), were more likely to answer knowledge questions correctly (risk reduction with statins 61% vs. 33%, p=.07; knowledge about options 57% vs. 33%, p=.002) and were more engaged by their clinicians in decision making (50. vs. 28, difference 21.4 (95% CI 6.4, 36.3), p=.01). We found no significant impact on patient satisfaction, medication starts, adherence or clinical outcomes, in part due to limited statistical power.

**Conclusion:**

DAs improved decisional outcomes without significant effect on clinical outcomes. DAs designed for point-of-care use with type 2 diabetes patients promoted shared decision making in nonacademic and rural primary care practices.

**Trial Registration:**

NCT01029288

## Background

Shared decision making (SDM) is a patient-centered approach to improve the quality of care of patients with diabetes and other chronic conditions [1]. Patient decision aids are tools designed to convey information about available options and their relative advantages and disadvantages to patients [2]. These decision aids, when designed for use during the consultation, create conversations that engage patients in making treatment decisions [3]. In close collaboration with a multidisciplinary team, including clinicians and patients with diabetes, we have developed decision aids to support the choice of antihyperglycemic agents and about using statins to reduce cardiovascular risk [3,4]. In efficacy randomized trials, these tools increased patient knowledge and engagement of patients in their treatment decisions [5-7]. In addition, self-reported medication adherence was improved in patients using the decision aid for statins [5].

While these trials provided evidence of efficacy of decision aids in patients with diabetes receiving care in academic clinics, little is known about the effectiveness of these tools in routine clinical practice, particularly in rural clinics. In this context of imperfect knowledge, state legislation in Washington and Minnesota and provisions in the Patient Protection and Affordable Care Act promote or require SDM [8,9]. The Center for Medicare and Medicaid Services evaluates accountable care organizations for their ability to implement SDM [10]. The National Quality Forum recommends measuring SDM as part of its framework for assessing quality of care in patients with multiple chronic conditions [11]. These legislative efforts require improvements in the evidence base about the effect of implementing SDM in usual clinical settings.

Thus, the objective of this study was to evaluate, in a cluster-randomized practical trial enrolling nonacademic and rural primary care practices and their patients with type 2 diabetes, the impact of patient decision aids vs. usual care on decision making measures, metabolic control and medication adherence.

We opted for a cluster-randomized trial, with randomization at the clinic level in order to reduce the risk of contamination, mitigate confounding with clinician communication style, and facilitate the implementation of study procedures and of the decision aid in each practice [12].

## Methods

We conducted a multicenter cluster randomized controlled trial set in nonacademic and rural primary care practices. These practices are affiliated either with Olmsted Medical Center (OMC) or the Mayo Clinic, and are located in Southeastern Minnesota, USA. Mayo Clinic and Olmsted Medical Center Institutional Review Boards approved the study protocol and all study procedures. These procedures appear in detail elsewhere [13] and we present these here briefly.

### Participants

Eligible participants were physicians, nurse practitioners, and physician assistants (i.e., clinicians) who cared for patients with type 2 diabetes at participating primary care practices. Practices were deemed eligible if they provided primary care for patients with type 2 diabetes. Minimal training was provided to clinicians that consented to participate [13]. Eligible patients were adults with >= 1 year of type 2 diabetes with a reason, identified by their clinician, to consider changing their antihyperglycemic or lipid-lowering regimens. For example, for the diabetes discussion, eligible patients had HbA_1C_ > 7.3, were not using insulin, and were not taking >2 antihyperglycemic agents at maximum dose. For the discussion about statins, eligible patients should not be using statins and should not have contraindications for taking statins. All patients and clinicians signed written informed consent for participation.

### Approach and protection against bias

**Figure 1:**
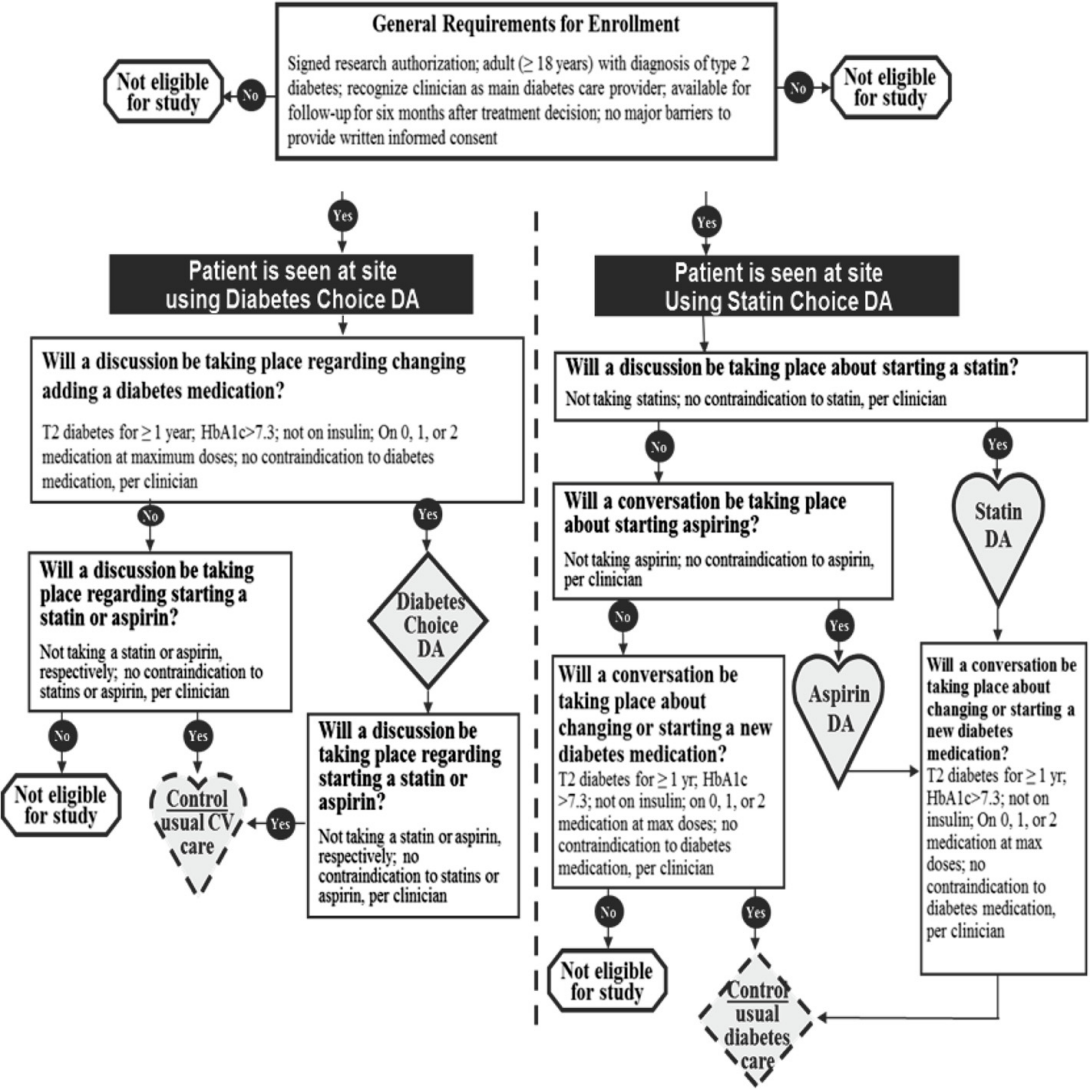
Study design for clinical trial.

Primary care practices were enrolled then matched by size (≤ 2 clinicians or > 2 clinicians) and randomly allocated by a statistician (who was the only team member aware of the composition of the pairs of practices) to i) the use of the *Diabetes Medication Choice* decision aid and usual care for lipid therapy medication (statin) discussion during the encounter or to ii) the use of the *Statin Choice* decision aid and usual care for antihyperglycemic medications discussion during the encounter [13]. This design allowed for each practice to incorporate a decision aid within their organization and for clinicians and their patients to qualify for either arm or both, while preventing contamination. Patients, who were kept unaware of the study hypotheses, and their clinician used the decision aids (http://shareddecisions.mayoclinic.org, Additional file 1) during the clinical encounter or proceeded with their encounter as usual (Figure 1).

### Data collection and outcomes

#### Decisional outcomes

We surveyed patients immediately after the clinical encounter with their clinician (and 3 and 6 months later) to ascertain their knowledge by assessing (a) how many of the 6 questions about the options and their pros and cons of antihyperglycemic agents patients answered correctly (Knowledge Transfer), and (b) how accurately they estimated their risk of having a heart attack within the next 10 years with and without statin use, in the statin group (Knowledge of Risk). Using the post-visit questionnaire, patients reported their decisional comfort by responding to the information, efficacy and satisfaction subscales of the Decisional Conflict Scale [14,15]. They also reported if a conversation about the pertinent medications took place, what decision was made about medications, and their satisfaction with the way in which information was shared.

We used a fidelity checklist created by our team to review video records of encounters to determine the extent to which clinicians were able to use the decision aid as intended in the decision aid arm or demonstrated similar behaviors in the control arm (reflecting potential contamination). The checklist included a minimum set (14 elements for the statin decision aid and 12 for the antihyperglycemic decision aid) that had to be present during the encounter. A pair of trained and calibrated investigators working independently and reproducibly (concordance correlation coefficient [16] = 0.95) evaluated video recorded encounters in intervention and control groups (when both clinicians and patients consented to video recording) and, using the OPTION score [17], assessed the extent to which the clinician was able to engage the patient in decision making about medications.

At the three- and six-month postal surveys, we asked patients about the implementation of their decision after the index encounter. The clinicians’ survey administered after the encounter inquired about what decision was made regarding medication during the encounter, and in the DA arm, the providers’ thoughts on the ease of use of the DA and the ease of incorporating it into practice.

#### Clinical outcomes

Medical records were reviewed at patient enrollment and at nine months post encounter. From the medical record, we extracted the patient’s medication list including medications added or changed during the index encounter and key laboratory parameters such as HbA1c and lipid profile. In patients who had a discussion about diabetes medications, we coded the HbA_1c_ at enrollment and six months post-enrollment as < 8%, 8-9% and >9%. Using the change in categories in this six-month period, we classified patients as having no change, a decrease, or an increase in glycemia. Similarly, we compared the change in LDL-cholesterol levels at six months vs. baseline, with categories drawn at below 5.55 mmol/L (100 mg/dL) or 5.55 mmol/L or greater, classifying patients as having had no change, an increase, or a decrease in LDL-cholesterol level.

We used pharmacy records for the 12-month period, starting 3 months prior to the study encounter and spanning up to nine months after it; to estimate adherence to medications added or changed during the index encounter. This estimate of adherence was calculated using the percentage of days covered (PDC) of 180 days post the clinical encounter with their clinician, defined as the number of days a patient had a supply of each medication divided by the number of days of eligibility for that medication. We also calculated the proportion of patients who were adherent (defined as PDC covered ≥80% of days) to that study drug throughout the follow-up period.

### Analyses

We planned to enroll 240 patients from 8 practices (30 patients per practice, 120 patients per arm) in order to have 90% power to detect a difference of 9.8 points in decisional comfort between the usual care arm and the decision aid arm with a significance level of 0.05 and assuming an intracluster correlation coefficient (ICC) of 0.05 [18]. Patient demographic characteristics were compared using a Fisher’s exact test for categorical variables and a Wilcoxon rank sum test for continuous variables. A regression model was used to adjust all continuous outcomes (decisional comfort post encounter and PDC) adjusting for both arm and type of discussion (diabetes medication or statins). We report adjusted means with 95% confidence intervals. For binary results (≥ 80% adherence), a logistic model was used to adjust for arm and type of discussion with odds ratios and 95% confidence intervals reported. The ICC’s were 0 or nearly so for all outcomes (knowledge, decisional comfort, medication adherence), indicating no clustering, so analyses were not adjusted for clustering. Furthermore, in a secondary analysis, all results were the same after adjusting for clustering (data not shown).

Secondary outcomes of satisfaction with knowledge transfer, change in laboratory values, and post-index visit cardiovascular risk knowledge (statin discussion only) were compared using a Fisher’s exact test. The post-index visit, 3 and 6 months knowledge (diabetes medication discussion only) was compared using the Wilcoxon rank sum test.

Study data were recorded and managed using the Research Electronic Data Capture (REDCap) system [19]. All analysis and data management were conducted using SAS (version 9.2) or Stata statistical software (version 11.0).

## Results

**Figure 2:**
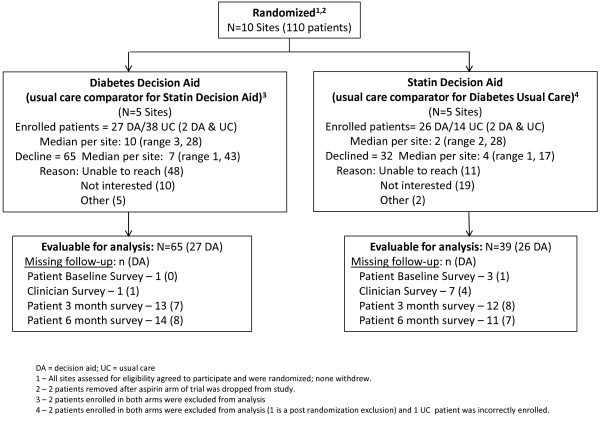
Flow diagram for clinical trial.

In the funded timeframe for this study, we could not enroll our target sample, but 110 patients were enrolled between April 2010 and July 2011 (Figure 2). We included 103 patients in the analysis, N=53 in the decision aid (DA) arm and N=50 in the usual care (UC) arm. We excluded 2 ineligible patients incorrectly enrolled (excluded without knowledge of their allocation or outcome [20]), and 2 patients enrolled to the usual care arm of the aspirin decision aid (an arm that was discontinued early in the trial due to poor feasibility without any patients allocated to the intervention arm [13]). In a protocol deviation and before examining the results, we also excluded 4 patients who were correctly allocated to both DA and UC; because we did not get enough patients in that situation to afford a separate analysis we present their results in Additional file 2.

All patient factors were well balanced across both arms with a difference found in the type of discussion that patients had (statins vs. diabetes medication; Table 1); subsequent results adjust for this difference. Forty-one clinicians participated in the trial, 27 participated in the diabetes medication group and 33 in the statin group (Table 2).

**Table 1:** Patient characteristics

***Character***	***Characteristics***	***Decision aid*(*N=53*)**	***Usual care*(*N=50*)**
		**n (%)**	**n (%)**
Discussion^1^	Diabetes medications	27 (51)	12 (24)
	Statin	26 (49)	38 (76)
Sex	Male	37 (70)	26 (52)
Age^2^: Mean (SD)		57.9 (10.5)	57.3 (11.4)
DM Duration	<5 years	24 (45)	29 (58)
	5+ years	29 (55)	21 (42)
Race^1^	White	53 (100)	36 (72)
	Other	0 (0)	3 (6)
	Unknown	0 (0)	11 (22)
Education^2^	HS or less	22 (44)	10 (22)
	Some college	19 (38)	25 (54)
	College +	9 (18)	11 (24)
Marital status^3^	Married	38 (78)	32 (68)
	Divorced/Separated/Widowed	7 (14)	9 (19)
	Never married	4 (8)	6 (13)
Income^3^	<40k	14 (30)	16 (37)
	40k or more	33 (70)	27 (63)
Health score^2^		76.0 (16.1)	74.0 (16.1)
BMI^2^		37.8 (8.1)	34.7 (10.1)
HbA1c^2,4^		8.7 (1.6)	8.0 (0.6)
	7-8	13 (48)	8 (67)
	>8	14 (52)	4 (33)
LDL^2,5^		103.2 (26.6)	105.2 (41.5)
	<100	12 (46)	18 (47)
Blood pressure	<130/80	30 (57)	30 (60)
MI Risk^2,5^		21.0 (12.0)	16.8 (12.4)

*Abbreviations*: *DM* Diabetes Mellitus, *HS* High School, *MI* Myocardial Infarction.

^1^p<0.05.

^2^Mean (Standard Deviation).

^3^Missing values not presented.

^4^Diabetes Medication discussion patients only.

^5^Statin Medication discussion patients only.

**Table 2:** Clinician characteristics

***Character***	***Characteristics***	***N*=*41 N*(%)**
Clinicians per site^1^		3.7, 2 (1, 14)
Arm	UC	25 (61)
	DA	35 (85)
Discussion	Diabetes medications	27 (66)
	Statin	33 (80)
Type of clinician^2^	Provider	27 (66%) [23]
	Resident/Fellow	7 (17%) [6]
	NP/PA	7 (17%) [6]
Gender	Male	24 (59)
Age^1,4^		44.6, 46.5 (28, 60)
Race	White	39 (95)
	Asian	1 (2.5)
	NH/PI	1 (2.5)
Years in practice^1,4^		14, 13.5 (1, 42)

*Abbreviations*: *NP* Nurse Practitioner, *PA* Physician Assistant, *NH* Native Hawaiian, *PI* Pacific Islander.

^1^Mean, Median (Range).

^2^N (%) [N that delivered DA]: 16 providers and 3 NP/PA delivered both DA and UC.

^3^Missing 9 responses.

^4^Missing 3 responses

### Decisional outcomes - patient outcomes

Decision aid use significantly increased knowledge transfer at baseline (Table 3). Patients were similarly satisfied with usual care and the decision aid; 71% of decision aid patients found the information provided helpful compared to 53% of patients in the usual care arm (p=.17) (Table 4). Patient decisional comfort was similarly high in both trial arms. Patients receiving the decision aid (diabetes medication or statin) were more likely to report having had a discussion to start or change a medication compared to those receiving usual care (77% vs. UC: 45%, P<.0001; Table 5), but there was no significant difference in the likelihood of start or change of a medicine in this group. In video-recorded encounters (N=39, 22 in the decision aid arm), clinician effort to engage patients in decision making was significantly higher in the decision aid arm with a mean difference of 21.4 points in the OPTION score (95% CI 6.4, 36.3). We found in video recorded visits that clinicians used the decision aid with moderate fidelity, completing on average 66% (95% CI 54, 78) of the checklist items. Usual care encounters covered 20% (95% CI 10, 29) of the items on the checklist, indicating minimal if any contamination (Table 5).

**Table 3:** Decisional outcome - knowledge assessment

	***Decision aid***	***Usual care***	***Mean diff*(*95*% *CI*)**
Knowledge transfer at baseline^1,2^, N	27	10	
Mean (95% CI)	56.8 (49.4, 64.2)	33.3 (20.8, 45.9)	23.5 (9.7, 37.3)
Knowledge transfer at 3 months^1,2^, N	20	8	
Mean (95% CI)	56.7 (47.4, 65.9)	50.0 (35.1, 64.9)	6.7 (−9.9, 23.2)
Knowledge transfer at 6 months^1,2^, N	19	8	
Mean (95% CI)	56.1 (48.5, 63.8)	54.2 (36.3, 72.0)	2.0 (−13.3, 17.3)
Knowledge of Risk w/out medication^3,4,5,6^	12 (52%)	16 (44%)	1.2 (0.7, 2.0)
Knowledge of Risk w/ medication^3,4,5,6^	14 (61%)	12 (33%)	1.8 (1.0, 3.2)

*p<0.05.

^1^Diabetes medication discussion only.

^2^% correct out of total asked (N=6 questions).

^3^Statin discussion only.

^4^Response to risk knowledge was correct.

^5^Missing 5 responses (DA=3, UC=2).

^6^Relative risk and 95% confidence interval.

**Table 4:** **Decisional outcome** - **satisfaction with knowledge transfer**^**1**,**2**^

	***Category***	***Decision aid*(*N=40*)**	***Usual care*(*N=21*)**	***p*-*value***^***3***^
Amount of information	Just right	32 (84%)	15 (75%)	0.50
	Too little	1 (3%)	2 (10%)	
	Too much	5 (13%)	3 (15%)	
Clarity of information	Extremely	22 (58%)	10 (50%)	0.45
	Somewhat	16 (42%)	9 (45%)	
	Not clear	0 (0%)	1 (5%)	
Helpfulness of information	Extremely	27 (71%)	10 (53%)	0.17
	Somewhat	11 (29%)	9 (45%)	
	Not helpful	0 (0%)	1 (5%)	
Would want for other decisions	For sure	22 (58%)	13 (65%)	0.30
	Not sure	16 (42%)	6 (30%)	
	Not at all	0 (0%)	1 (5%)	
Recommend to others	Strongly for	24 (63%)	12 (60%)	>.99
	Not sure	14 (37%)	8 (40%)	
	Strongly against	0 (0%)	0 (0%)	

^1^Of those that felt a discussion took place.

^2^Missing responses: DA=2, UC=1.

^3^Fisher exact test statistic p-value.

**Table 5:** **Decisional outcome** - **decision and comfort with decision**

	***Decision aid***	***Usual care***	***Relative risk* (*95*%*CI*)**
Had a discussion about starting or changing a medication	40 (77%)	21 (45%)	1.8 (1.2, 2.5)
Decided to start/change a medication^1^	13 (33%)	9 (43%)	0.8 (0.4, 1.5)
			***Mean difference (95% CI)***
Comfort of decision (DCS)^1,2^	82.9 (78.8, 87.0)	81.2 (75.3, 87.1)	1.7 (−5.3, 8.7)
Perception of knowledge (DCS subscale)^1,2^	80.5 (75.1, 85.9)	74.9 (67.1, 82.6)	5.6 (−3.6, 14.8)
Adequacy of support (DCS subscale)^1,2^	80.9 (76.1, 85.8)	81.0 (74.1, 87.9)	−0.05 (−8.3, 8.2)
Effect (DCS subscale)^1,2^	86.4 (82.3, 90.5)	86.1 (80.2, 91.9)	0.29 (−6.7, 7.3)
Level of patient engagement (OPTION)^2,3^	49.7 (40.6, 58.7)	28.3 (15.1, 41.6)	21.4 (6.4, 36.3)
Fidelity^2,3^	65.7% (53.8, 77.6)	19.6% (9.9, 29.3)	46.1% (30.3, 61.8)

^1^Of those that had a discussion.

^2^Adjusted Mean (95% CI). Regression model of arm and type of discussion (diabetes medication vs. statin).

^3^39 patients that consented to video analysis (DA=22, UC=17).

### Decisional outcomes - clinician outcomes

Clinicians using the decision aids were more likely to report having had a conversation about starting or changing a medication with the patient (Table 6). There was greater agreement amongst patients and clinicians using decision aids on whether a conversation occurred or not regarding medications, but the difference in the frequency of concordance was not significant (74% vs. 64%, p=.30). Clinicians that administered the decision aids found them easy to very easy to use (80%), and 80% reported that it was easy to very easy for their staff to integrate decision aids into the practice workflow.

**Table 6:** **Decisional outcome** - **primary care clinician results**

	***Decision aid* (*N*=*49*)**	***Usual care*(*N*=*47*)**	***Relative risk* (*95*%*CI*)**
Had discussion about starting or changing a medication	44 (90%)	32 (68%)	1.3 (1.1, 1.6)
Decided to start/change a medication^1^	14 (32%)	6 (19%)	1.7 (0.7, 4.0)
Agreement between clinician and patient on having a discussion^1^	34 (74%)	23 (64%)	1.2 (0.9, 1.6)
Clinician view of DA	N=40	~	
Delivery of DA was:			
Easy to very Easy	32 (80%)	~	
Neither	4 (10%)	~	
Difficult to very difficult	4 (10%)	~	
Integration of DA was^2^:			
Easy to very easy	32 (80%)	~	
Neither	7 (18%)	~	
Difficult to very difficult	0 (0%)	~	

^1^Of those that had a discussion.

^2^Missing 1 response.

### Clinical outcomes

There was no difference in the proportion of patients achieving improved glycemic control in the decision aid or usual care arm (44% vs. 50%, p=.25); both arms achieved similar levels of LDL cholesterol (Table 7). Twenty five out of 30 (83%) patients within the decision aid arm that received a prescription filled the prescription (Table 7), compared to 22 of 22 patients (100%) in the usual care arm. The fill rate for statins was 100% in both arms; all non-starts were seen within the patients that had a diabetes medication discussion. No difference was found in PDC overall or within each discussion group. In the statin arm of the trial, we found no significant association between patient estimated risk and rate of statin prescription.

**Table 7:** **Clinical outcomes** - **lab results and medication adherence**

	***Decision aid***	***Usual care***	***p value***^***5***^
*Lab results*			
HbA1c^1^			0.25
No change	12 (48%)	3 (25%)	
Increase	2 (8%)	3 (25%)	
Decrease	11 (44%)	6 (50%)	
LDL^2^			0.57
No change	13 (68%)	14 (52%)	
Increase	1 (5%)	2 (7%)	
Decrease	5 (26%)	11 (41%)	
*Medication adherence results*			
Decided to start or change a medication	13 (33%)	9 (43%)	0.58
Prescription data was obtained	43 (81%)	44 (88%)	
Has a prescription for medication	30 (57%)	22 (44%)	
Diabetes	25 (83%)	11 (50%)	
Statin	5 (17%)	11 (50%)	
Filled prescription for medication	25 (83%)	22 (100%)	
Diabetes	20 (80%)	11 (100%)	
Statin	5 (100%)	11 (100%)	
# of medications filled	49	28	
Diabetes^3^	44	17	
Statin	5	11	
Percentage of days covered ≥ 80%	38 (78%)	22 (79%)	>.99
Diabetes	34 (77%)	15 (88%)	0.48
Statin	4 (80%)	7 (64%)	>.99
Percentage of days covered ≥ 80%^4^	1.44 (0.5, 4.1)	1 (Ref.)	0.49

^1^Diabetes medication discussion patients only.

^2^Statin medication discussion patients only.

^3^Patients taking multiple diabetes medications.

^4^Odds ratio (95% CI), logistic model of discussion and arm.

^5^Fisher’s exact test statistic (unless noted otherwise).

## Discussion

### Our findings

Compared to usual care, patients participating in encounters using decision aids were more likely to report participating in a conversation about medications, gained more knowledge about the options, and were more likely to have their clinician put effort into engaging them in the decision making process. There was no significant impact on patient satisfaction, on choice of starting or adhering to medicines and on measures of metabolic control; these estimates, though, were imprecise due to low statistical power.

### Limitations and strengths

This trial was smaller than planned mostly due to challenges in the timely recruitment of clinicians and practices; this reduced the precision with which we were able to estimate the intervention effect on clinical outcomes. We were able to obtain video recordings from 38% of encounters. This limits our ability to use our checklist and to obtain an OPTION score in all encounters thus reducing our confidence in the inferences related to fidelity and clinicians’ efforts to engage patients in decision making, respectively. Clinicians and staff of rural primary care clinics generously engaged in this study, seeking with the study team to overcome challenges related to distance and sparse eligible patients. The infrequent opportunity to use the decision aids coupled with our tactic to provide minimal training to clinicians in the use of the decision aids led to only a few patients receiving the decision aid as intended (~30% of encounters covered half or less of the fidelity items). While we did not appreciate important clustering effects, our trial has all the challenges of clustered-randomized trials in that the small number of clusters reduces the likelihood that randomization will achieve balanced prognosis. Also, incomplete recruitment of clinicians and patients within each cluster might introduce selection bias, particularly through incomplete allocation concealment once sites have been allocated but clinicians and patients are still being recruited.

On the other hand, our inferences are strengthened by concealed randomization of practices and blinding of patients (to the hypotheses of the study), data collectors and analysts (to allocation), and by protecting the results from confounding by contamination and poor fidelity. The consistency of our findings here with findings in efficacy trials, particularly on decisional outcomes, further strengthens our inferences. The effects of SDM on clinical outcomes, however, remain unclear.

### Comparison with prior research

This study, along with others we have conducted, has found improvements in knowledge, decisional comfort, and patient participation in decision making, and little impact on adherence and other patient health outcomes. The Diabetes Medication Choice study [6] found a significant increase in knowledge over usual care (adjusted mean difference 1.10 (95% CI 0.11, 2.09), an increase of patient involvement on average of 21.8 of 100 points, but no impact on medication adherence at 6 months nor an impact on HbA1c levels. The first Statin Choice randomized trial [5] conducted in a specialty setting found that patients who received the decision aid were 22.4 times more likely to know their estimated cardiovascular risk than those in the usual care group, had greater decisional comfort (10.6 points higher on a 100 point scale), and better self-reported adherence at 3 months, odds ratio 3.4 (95% CI 1.5, 7.5). The second Statin Choice clinical trial [7] conducted by another group found an increase in patient knowledge of their cardiovascular risk as well (odds ratio 1.9, 95% CI 1.0, 3.8), an increase in decisional comfort among the informed subscale and support subscale, and no difference in medication adherence at six months. Our findings, while imprecise, are consistent with these prior results suggesting it is feasible to observe similar outcomes in academic and nonacademic practices, provided that the decision aids are implemented.

### Implications for policy, practice and research

We have been able to conduct trials of decision aids for use during the consultations of people with diabetes in academic subspecialty and primary care and, in this study, in nonacademic and rural primary care settings. In these contexts, the decision aids have been well received by patients and clinicians, and have improved decisional outcomes. This effectiveness has not necessarily translated into a favorable impact on clinical measures of effect or on medication adherence. We have seen this not only here, but also in our prior studies and on the recent Cochrane review of decision aids [2]. This might result from two closely related issues. The first issue is that decision aids that operate during the consultation require attending the consultation, itself a manifestation of adherence. Thus, without invoking trial selection bias, the requirement for our decision aids to operate in the consultation may limit their exposure to non-adherent patients. This limits the opportunity to find effects on medication adherence. The second closely related issue is that shared decision making is appropriate when there is more than one sensible management option. When one option yields superior outcomes with acceptable harms and costs, then most patients will select this option, and the outcomes achieved by the group will reflect the effect of each patient implementing that option. Any variability observed across patients will result from chance, differences in biological response, and treatment adherence. When more than one option is acceptable, however, the effect of different patients opting for different treatments will contribute to the overall variability in outcomes. Therefore, in the absence of a superior treatment choice, the only way shared decision making can improve patient outcomes is through improvements in adherence to the selected treatment option. Thus, there is no a priori reason to believe that shared decision making should improve clinical outcomes beyond its effect on adherence to treatment.

Research to select patients most likely to benefit from SDM and to explore ways to improve high-fidelity delivery of the tools may improve their clinical utility. We have discussed above that the observed fidelity of use could suggest that some effectiveness might not have been realized because of insufficient clinician training. An alternative explanation is that patient-centered care sometimes necessitates deviation from the expected use of the tools in order to accommodate emerging patient issues. Whether this means that more effort to improve optimal use of the tools is necessary warrants further exploration.

Despite the uncertainty about their impact on clinical outcomes, the value of decision aids as promoters of patient-centered practice and patient engagement remains, in our view, the most important justification for their use. DAs promote patient-centered practice to the extent that they support both parties in having an evidence-based discussion in which patient participation in deliberation is dynamically and empathically negotiated by the parties. DAs do not guarantee patient-centered care to the extent that the practices, norms, rituals, and policies of the practice may fail to support it [21]. Similarly, patient engagement is facilitated by the common ground offered by the decision aid, but it might not happen if the patient is not in a position to participate or feels threatened by such participation [22]. Thus, DAs are tools to promote and facilitate participatory forms of decision making, but much work is needed to increase the likelihood that shared decision making results from their use.

We do not think the results from this and past trials will satisfy those pursuing legislation of SDM seeking reductions in healthcare utilization and costs [23]. To this extent, the Patient Protection and Affordable Care Act of 2010 did not fund, but promoted the establishment of SDM Resource Centers. The act also created the Patient-Centered Outcomes Research Institute (PCORI) that funds comparative effectiveness research and communication of evidence through SDM [9]. In our view, current federal policy strikes the right balance, supporting additional research rather than mandating implementation. State legislatures, however, have been more sanguine in their approach [8]. Clearly large studies outside of academia will be needed to know the full extent of intended and unintended consequences of SDM.

## Conclusion

This small trial to examine the effectiveness of decision aids in nonacademic and rural clinical practices revealed (a) difficulties in recruitment of clinicians (and their patients), (b) a need to train and support clinicians in the use of the decision aids, (c) that using decision aids is indeed feasible in the care of patients with type 2 diabetes in these clinics, and (d) point-of-care decision aids are effective in improving the quality of decision making in the care of this patients.

## Abbreviations

(SDM): Shared decision making; (OMC): Olmsted medical center; (DA arm): Decision aid arm; (PDC): Percentage of days covered; (ICC): Intracluster correlation coefficient; (REDCap): Research Electronic Data Capture; (PCORI): Patient-Centered Outcomes Research Institute.

## Competing interests

The authors declare that they have no competing interests.

## Authors’ contributions

MB, drafted manuscript, participated in the design of the study and performed the statistical analysis. AL, managed the conduct of the study, helped draft the manuscript. NS, participated in the design of the study, participated in the draft of the manuscript. VM, conceived the study, participated in the design of the study, participated in the draft of the manuscript. KR, managed the study and participated in the draft of the manuscript, KT, participated in the design of the study and in the draft of the manuscript, BY, conceived the study, participated in the design of the study. MK, managed the conduct of the study. HVH, performed the statistical analysis, helped with the draft of the manuscript. LP, managed the conduct of the study and enrolled patients. All authors read and approved the final manuscript.

## Pre-publication history

The pre-publication history for this paper can be accessed here:

http://www.biomedcentral.com/1472-6963/13/301/prepub

## Supplementary Material

Additional file 1 Pictorial of Diabetes Choice Decision Aid. Click here for file

Additional file 2 Patients excluded from analysis. Click here for file
