# Supplementary material for: Shared decision making for patients with type 2 diabetes: a randomized trial in primary care
Source: BMC Health Serv Res. 2013 Aug 8;13:301. doi: 10.1186/1472-6963-13-301 (PMC3751736; doi:10.1186/1472-6963-13-301)
Supplement: Additional file 1 — Pictorial of Diabetes Choice Decision Aid. [file 1472-6963-13-301-S1.pdf]

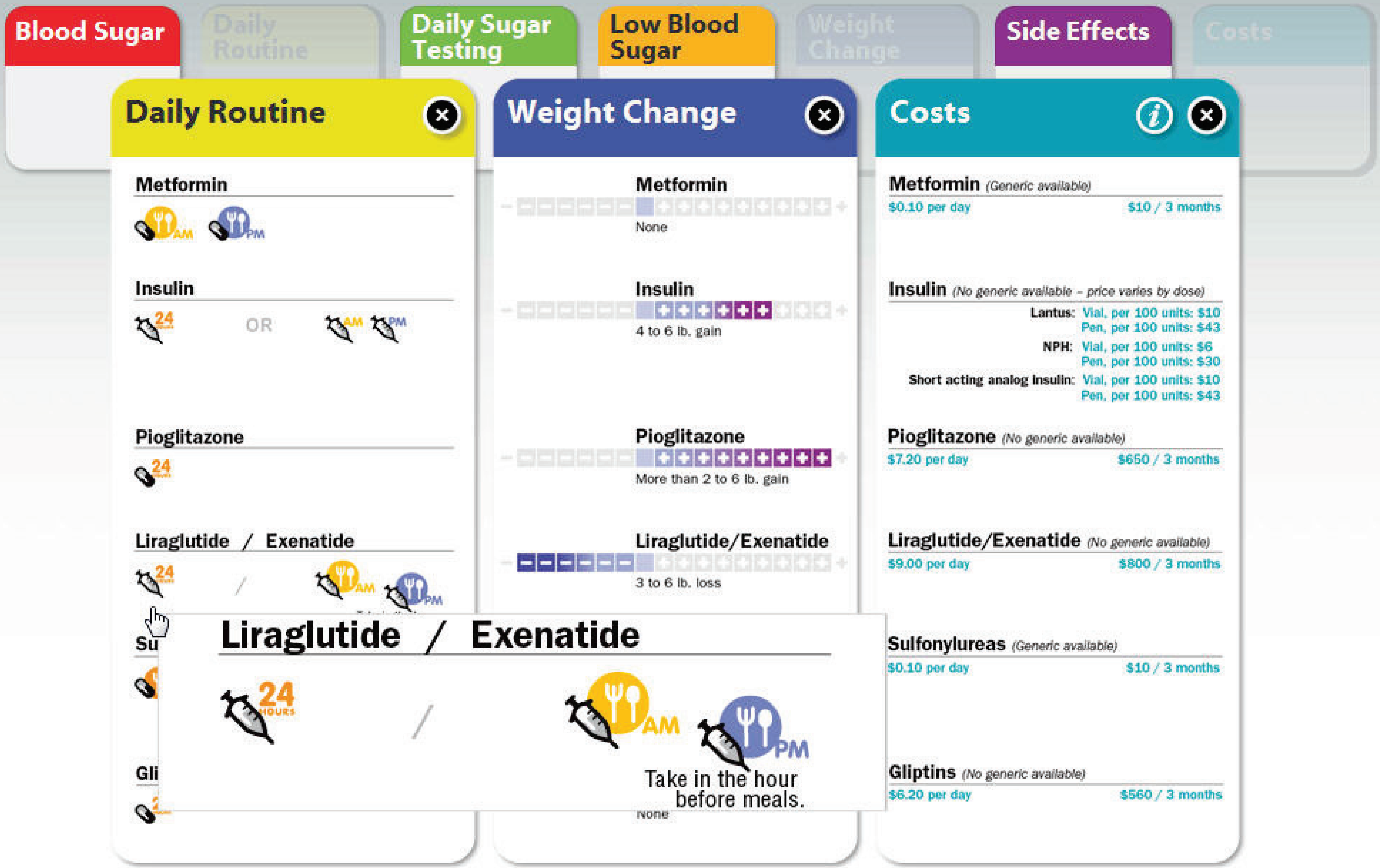

Diabetes Decision Aid

1 What is my risk of having a heart attack in the next 10 years?

The risk for 100 people like you who **DO NOT** take statins.

NO STATIN

50 people **DO NOT** have a heart attack (green)

50 people **DO** have a heart attack (red)

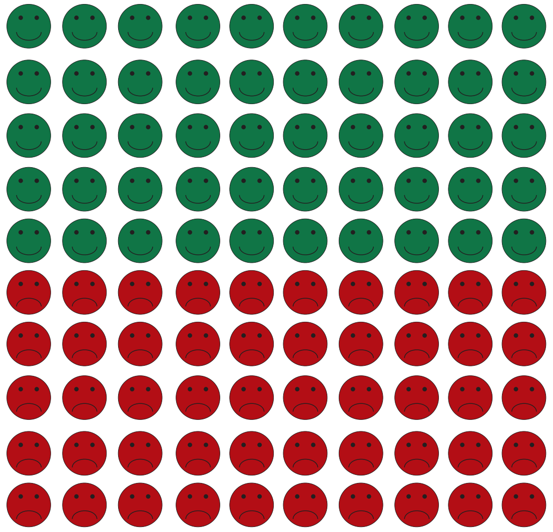

The risk for 100 people like you who **DO** take statins.

YES STATIN

50 people still **DO NOT** have a heart attack (green)

12 people **AVOIDED** a heart attack (yellow)

38 people still **DO** have a heart attack (red)

88 people experienced **NO BENEFIT** from taking statins

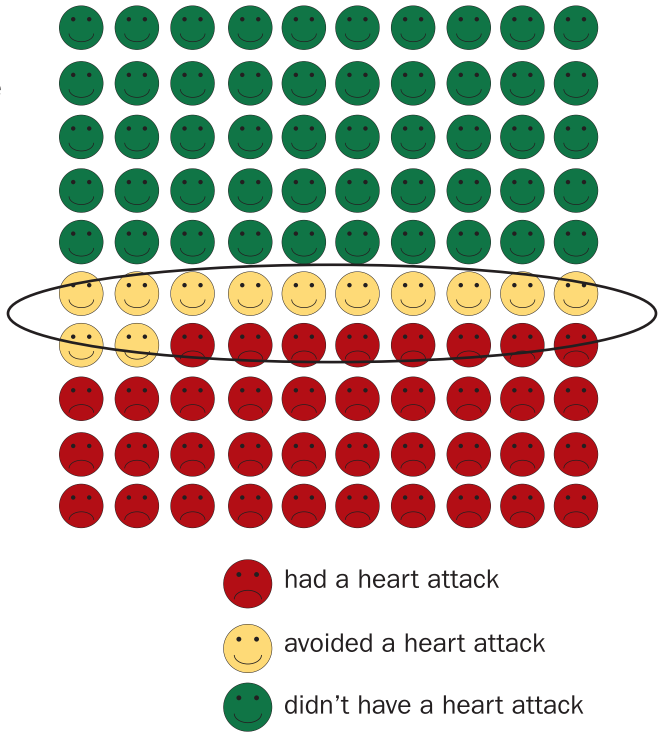

2 What are the downsides of taking statins (cholesterol pill)?

- Statins need to be ***taken every day*** for a long time (maybe forever).
- Statins cost money. (to you or your drug plan)
- **Common side effects:** nausea, diarrhea, constipation (most patients can tolerate)
- **Muscle aching/stiffness:** 5 in 100 patients (some need to stop statins because of this)
- **Liver blood test goes up** (no pain, no permanent liver damage): 2 in 100 patients (some need to stop statins because of this)
- **Muscle and kidney damage:** 1 in 20,000 patients (requires patients to stop statins)

3 What do you want to do now?

- ☐ Take (or continue to take) statins
- ☐ Not take (or stop taking) statins
- ☐ Prefer to decide at some other time

HIGH (>30%) | © 2010 Mayo Foundation for Education and Research. All Rights Reserved.
