# Supplementary material for: Shared decision making for patients with type 2 diabetes: a randomized trial in primary care
Source: BMC Health Serv Res. 2013 Aug 8;13:301. doi: 10.1186/1472-6963-13-301 (PMC3751736; doi:10.1186/1472-6963-13-301)
Supplement: Additional file 2 — Patients excluded from analysis. [file 1472-6963-13-301-S2.pdf]

Appendix B: Patients excluded from analysis

	Patient 1	Patient 2	Patient 3*	Patient 4	Patient 5	Patient 6
Discussion & Arm						
Statin DA/Diabetes UC	X		X			
Statin UC/Diabetes DA		X		X		
Aspirin UC					X	X
Age ≤ 65	Yes	Yes	Yes	Yes	Yes	No
Race	White	White	Other	White	White	White
Education	Some College	Some College	Some College	Some College	Some College	4 Yr College
Marital Status	Married	Married	Married	Married	Married	Married
Income	<40k	40k+	40k+	40k+	40k+	40k+
Years w/ diabetes	5+	5+	5+	<5	<5	5+
MI Risk	24%	~	9%	~	5.6%	23.6%
HbA1c	8.5	8.3	7.6	8.5	6.1	6.2
LDL	58	101	93		142	87
Blood Pressure	117/73	127/75	124/82	136-96	116/66	110/62
Outcomes:						
Had a discussion about starting or changing a medication	Yes for Diabetes & Statin	Yes for Diabetes/ No response for Statin	Yes for Diabetes & Statin	Yes for Diabetes/ No for statin	Yes	No
Start medication	Yes for Diabetes & Statin	Yes	Yes for Diabetes/ No for Statin	Yes	No	~
Knowledge Diabetes at Baseline	2/6	6/6	2/6	5/6	~	~
Knowledge of Risk w/out medication at Baseline	Correct	~	Correct			
Knowledge of risk w/ medication at baseline	15 or so	~	8 or so			
DCS	100	92.5	100	100	75	~
Amount of Information	Just right	Missing	Just right	Missing	Missing	Missing
Clarity of Information	Somewhat	Missing	Extremely	Missing	Missing	Missing
Helpfulness of Information	Extremely	Missing	Extremely	Missing	Missing	Missing
Would want for other decisions	For Sure	Missing	For Sure	Missing	Missing	Missing
Recommend to others	Strongly for	Missing	Not Sure	Missing	Missing	Missing

* Post randomization exclusion
